# Supplementary material for: Predicting Variation of DNA Shape Preferences in Protein-DNA Interaction in Cancer Cells with a New Biophysical Model
Source: Genes (Basel). 2017 Sep 18;8(9):233. doi: 10.3390/genes8090233 (PMC5615366; doi:10.3390/genes8090233)
Supplement: Supplementary file 1 [file genes-08-00233-s001.docx]

**Predicting variation of DNA shape preferences in protein-DNA interaction in cancer cells with a new biophysical model**

**Kirill Batmanov^1^ and Junbai Wang^1,*^**

1. Department of Pathology, Oslo University Hospital – Norwegian Radium Hospital, Montebello 0310, Oslo, Norway

*. Correspondence to [junbai.wang@rr-research.no]

**Supplementary Information**

**Supplementary Methods**

**Hyperparameter update formulae**

Here we present more technical details about the hyperparameter update step in the model fitting.

When computing the Hessian matrix $H$ during the hyperparameter update of the shape model, we use the fact that the shape model data cost function can be represented as the full dinucleotide cost function $L_{D}^{\text{dinuc}}$ with dinucleotide parameters linearly transformed:

$$\begin{aligned} L_{D}\left( w, d^{f},\mu,a,b \right)=L_{D}^{\text{dinuc}}\left( w,d,\mu,a,b \right) (1) \end{aligned}$$

$$d=d^{f}\cdot D$$

$L_{D}^{\text{dinuc}}$ was called $E(S)$ in (1), and its Hessian matrix $H^{\text{dinuc}}\left( w,d,\mu,a,b \right)$ can be computed in a straightforward way using the R-propagation algorithm (2). Then $H$ can be computed by transforming appropriate parts of $H^{\text{dinuc}}$ in the following manner:

$$\frac{\partial^{2}L_{D}}{\partial x \partial d_{p,k}^{f}}=\sum_{i=1}^{16} D_{k,i}\frac{\partial^{2}L_{D}^{\text{dinuc}}}{\partial x \partial d_{p,i}},\text{ for }x\in\left\{ w_{j,a},\mu,a,b \right\}$$

$$\frac{\partial^{2}L_{D}}{\partial d_{p_{1},k_{1}}^{f} \partial d_{p_{2},k_{2}}^{f}}=\sum_{i=1}^{16} \sum_{j=1}^{16} D_{k_{1},i}D_{k_{2},j}\frac{\partial^{2}L_{D}^{\text{dinuc}}}{\partial d_{p_{1},i} \partial d_{p_{2},i}}$$

These expressions are derived from equation (1). Second derivatives of the full dinucleotide model ($\frac{\partial^{2}L_{D}^{\text{dinuc}}}{\partial x \partial d_{p,i}}$ and $\frac{\partial^{2}L_{D}^{\text{dinuc}}}{\partial d_{p_{1},i} \partial d_{p_{2},i}}$) are computed by the R-propagation algorithm in the same way as in (1). When several model weights $W_{i_{1}},\ldots,W_{i_{n}}$ share a single regularization hyperparameter $\alpha_{m}$, the update formula for this alpha is as follows:

$$\alpha_{m}=\frac{\gamma_{m}}{\sum_{k=1}^{n} W_{i_{k}}}$$

$$\gamma_{m}=\sum_{k=1}^{n} 1-\alpha_{m}H_{i_{k}i_{k}}^{-1}=n-\alpha_{m}\sum_{k=1}^{n} H_{i_{k}i_{k}}^{-1}$$

The update for $\beta$ is done according to the formulae in the main text.

**Supplementary Figures**

**Supplementary Figure S1. Fitted shape preferences of MAX and BHLHE40 for different cell lines (positive part).**

The heatmaps show the preference for each shape feature at each position (the inferred $d^{f}$ matrix). Only the positive preferences are shown, that is, the preferences towards lower values of shape features. Colours faded to grey mean weaker preference. The PWM logos are shown above the shape preferences heatmaps.

**
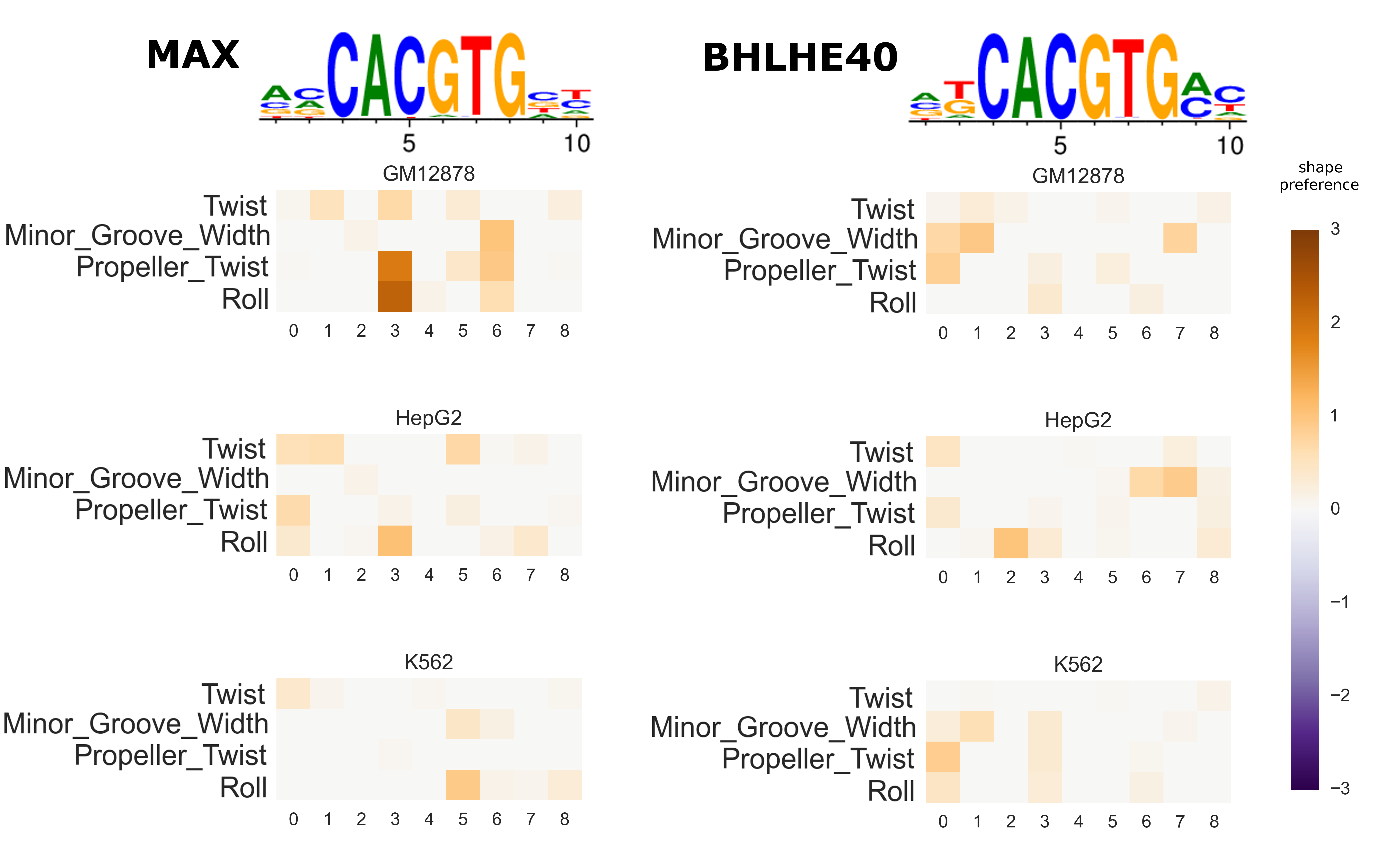
**

**Supplementary Figure S2. Shape model parameters in different conditions (negative part).**

The heatmaps show the preference for each shape feature at each position (the inferred $d^{f}$ matrix). Only the negative preferences are shown, that is, the preferences towards lower values of shape features. Colours faded to grey mean weaker preference. The PWM logos are shown above the shape preferences heatmaps.

**
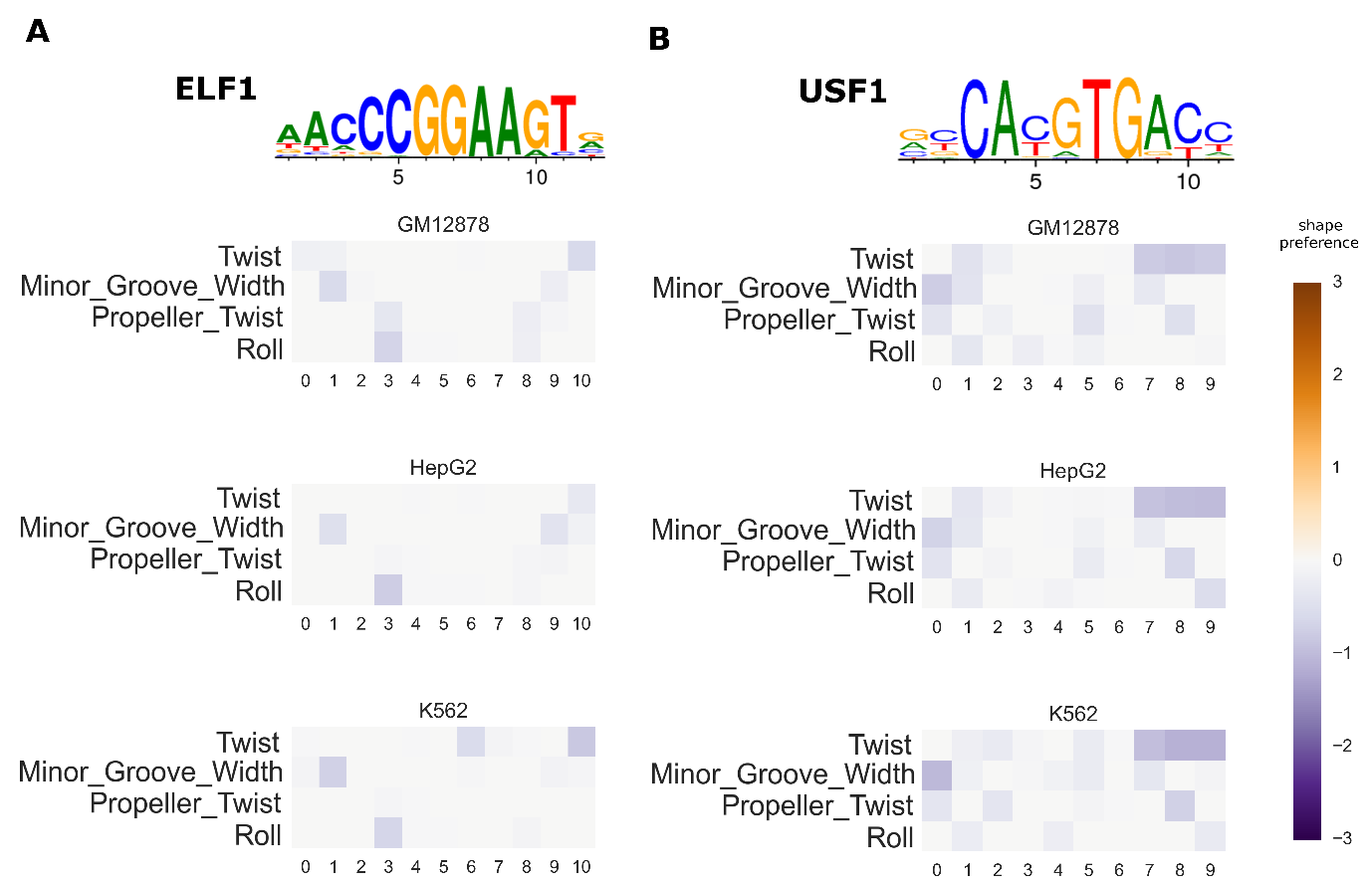
**

**Supplementary Figure S3. Changes in shape preferences.**

**A.** Variation of inferred shape preferences of ERα at different times after treatment with E2 in MCF7 cell line. **B.** Variation of inferred shape preferences of FOXA1 after treatment with either Dex or E2 in MCF7 cell line. The heatmaps show the preference for each shape feature at each position (the inferred $d^{f}$ matrix). Only the negative preferences are shown in **A**, and only the positive preferences in **B**. Colours faded to grey mean weaker preference. The PWM logos are shown above the shape preferences heatmaps.

**
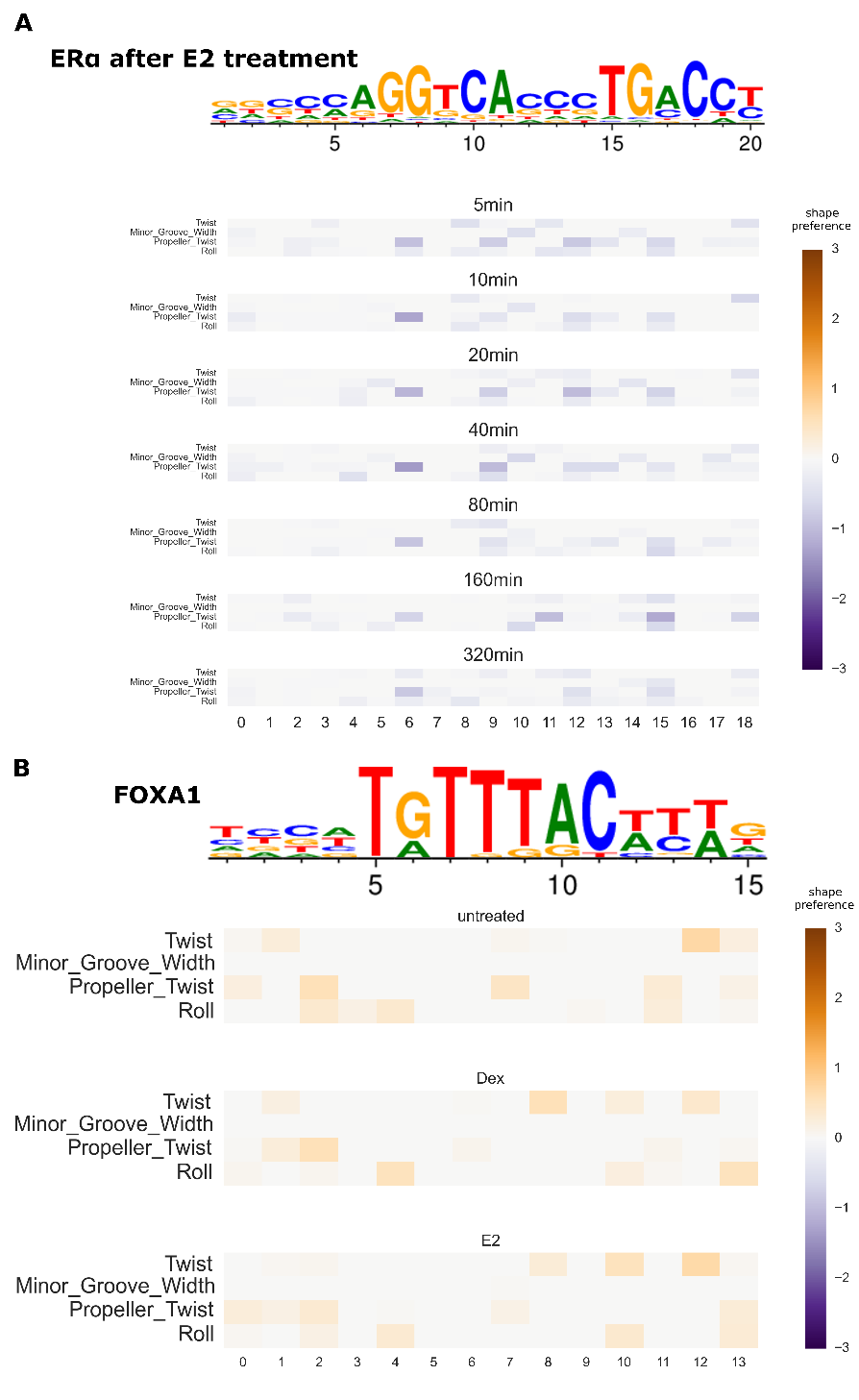
**

**Supplementary Figure S4. ASB prediction of the full dinucleotide and shape-restricted models compared to the independent model.**

For each of the 36 ASB datasets, AUPRC of the independent model is compared to **A)** the full dinucleotide model, and **B)** the DNA shape-restricted dinucleotide model. The P value of the two sided Wilcoxon signed rank test comparing the independent and dependent AUPRC (n = 36) is given.

**
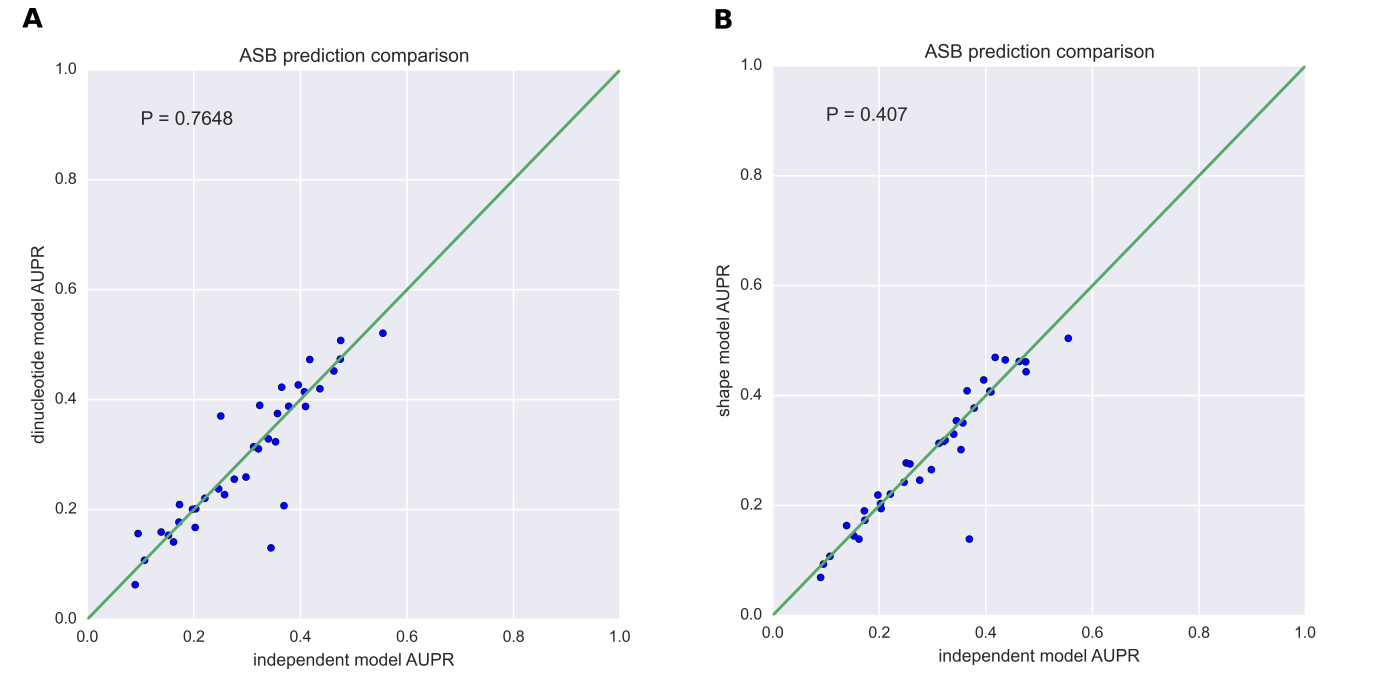
**

**Supplementary references**

1. Wang, J. (2014) Quality versus accuracy: result of a reanalysis of protein-binding microarrays from the DREAM5 challenge by using BayesPI2 including dinucleotide interdependence. *BMC bioinformatics*, **15**, 289.

2. Wang, J. and Morigen. (2009) BayesPI - a new model to study protein-DNA interactions: a case study of condition-specific protein binding parameters for Yeast transcription factors. *BMC bioinformatics*, **10**, 345.
